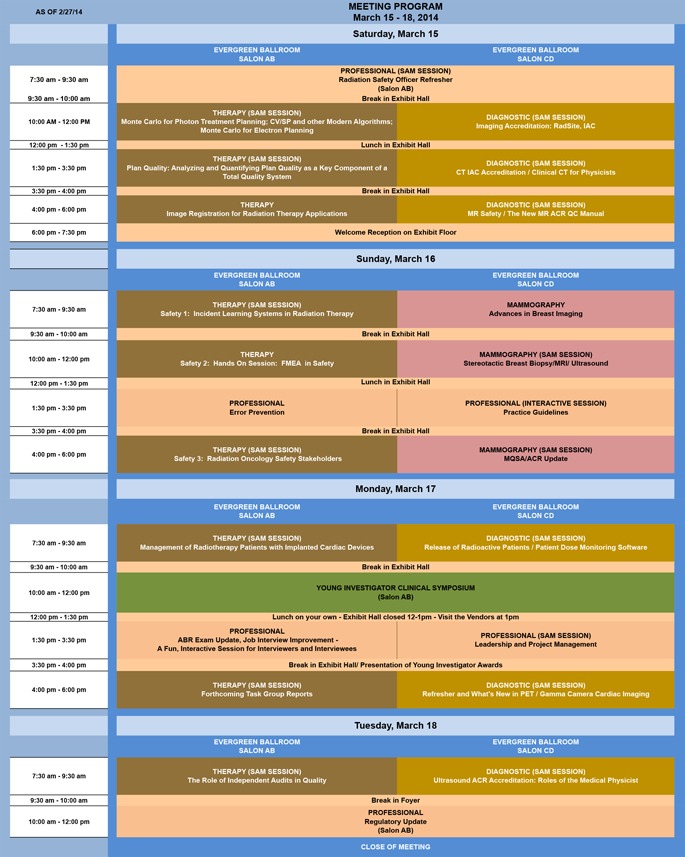# MEETING PROGRAM

**DOI:** 10.1120/jacmp.v15i3.5054

**Published:** 2014-04-29

Available on‐line at www.aapm.org/meetings/2014SCM/

2014 AAPM Spring Clinical Meeting March 15 ‐ 18, 2014 Denver, CO

**Chair**

Brian Wang, PhD

University Louisville

Louisville, KY

**Organizers**

**THERAPY TRACK**

Jean M. Moran, PhD

Dept of Radiation Oncology B2C438

Ann Arbor, MI

Dimitris Mihailidis, PhD

Rad Onc and Med Phys, Charleston Radiation Therapy Cons

Charleston, WV

**PROFESSIONAL TRACK**

Jessica B. Clements, MS

Medical Physics, Texas Health Presbyterian Hospital Dallas

Dallas, TX

Michael Howard, PhD

Sarah Cannon Cancer Center, Parkridge Medical Center

Chattanooga, TN

**DIAGNOSTIC TRACK**

Robert A. Pooley, PhD

Radiology, Mayo Clinic

Jacksonville, FL

Dustin Gress, MD

MD Anderson Cancer Center

Houston, TX

**YOUNG INVESTIGATOR PROGRAM**

Jessica B. Clements, MS

Medical Physics, Texas Health Presbyterian Hospital Dallas

Dallas, TX

Brian Wang, PhD

University Louisville

Louisville, KY

David E. Hintenlang, PhD

University of Florida

Gainesville, FL

**MAMMOGRAPHY TRACK**

William Geiser, MS

Imaging Physics, M.D. Anderson Cancer Center

Houston, TX

Jean M. Moran, PhD

Dept of Radiation Oncology B2C438

Ann Arbor, MI

**Table 1:**